# Agenesis of maxillary lateral incisor in an Angle Class II, Division 1 malocclusion patient

**DOI:** 10.1590/2177-6709.20.5.108-117.bbo

**Published:** 2015

**Authors:** Guilherme Thiesen

**Affiliations:** 1 Professor of Orthodontics, Universidade do Sul de Santa Catarina (UNISUL), Florianópolis, Santa Catarina, Brazil. Certified by the Brazilian Board of Orthodontics and Dentofacial Orthopedics (BBO).

**Keywords:** Anodontia, Angle Class II malocclusion, Corrective Orthodontics

## Abstract

The present case report describes the orthodontic treatment of a patient with agenesis of maxillary left lateral incisor and Angle Class II, Division 1 malocclusion. The patient also presented with maxillary midline deviation and inclination of the occlusal plane in the anterior region. Treatment objectives were: correction of sagittal relationship between the maxilla and the mandible; correction of midline deviation, so as to cause maxillary and mandibular midlines to coincide; correction of overbite and leveling of the occlusal plane, so as to create ideal conditions for esthetic rehabilitation of anterior teeth. This case was presented to the Brazilian Board of Orthodontics and Dentofacial Orthopedics (BBO) as a requirement for the title of certified by the BBO.

## Abstract (pt)

O presente caso clínico relata o tratamento ortodôntico de um paciente portador de agenesia do incisivo lateral superior esquerdo e má oclusão de Classe II 1^a^ divisão de Angle. Apresentava, ainda, desvio da linha média superior e inclinação do plano oclusal na região anterior. Os objetivos do tratamento foram adequar a relação sagital entre as arcadas, proporcionar a coincidência entre as linhas médias superior e inferior, corrigir a sobremordida e nivelar o plano oclusal, criando condições apropriadas para a reabilitação estética dos dentes anteriores. Este caso foi apresentado à Diretoria do Board Brasileiro de Ortodontia e Ortopedia Facial (BBO) como parte dos requisitos para a obtenção do título de Diplomado pelo BBO.

## INTRODUCTION

A 26-year and 5-month-old male patient sought orthodontic treatment in good general health and with occasional smoking habit. His chief complaint was "impaired esthetics in the anterior region." His dental history reported trauma of tooth #11 suffered five years before, when he was subject to endodontic treatment. The patient presented with dark discoloration of the clinical crown. He had also been subject to esthetic rehabilitation of tooth #23 with dental composite and stripping, and presented with adequate oral health. Functionally, the patient presented lateral disocclusion through molar guidance on the left side. Mouth opening and closure movements were performed without deviation, with the temporomandibular joint free of any symptoms.

## DIAGNOSIS

**Figure 1:**
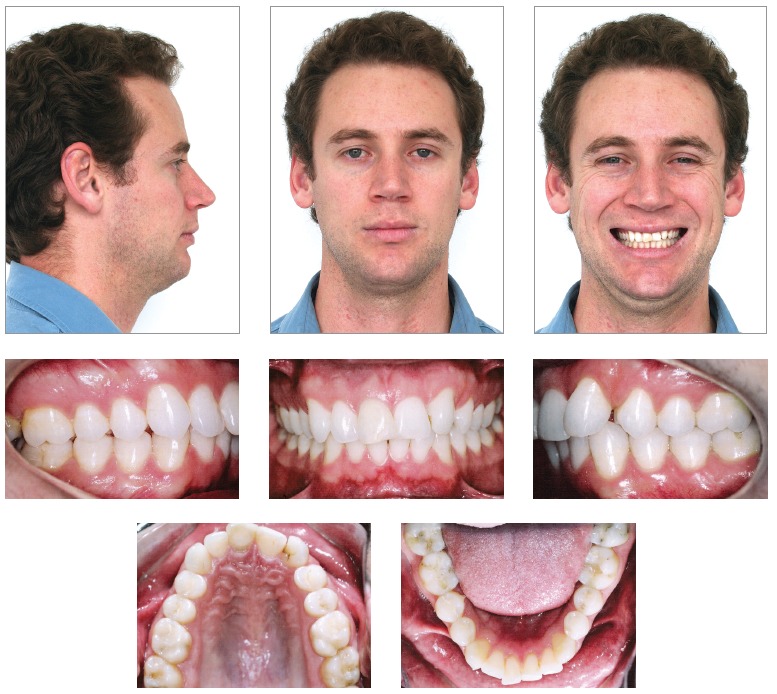
- Initial facial and intraoral photographs.

**Figure 2:**
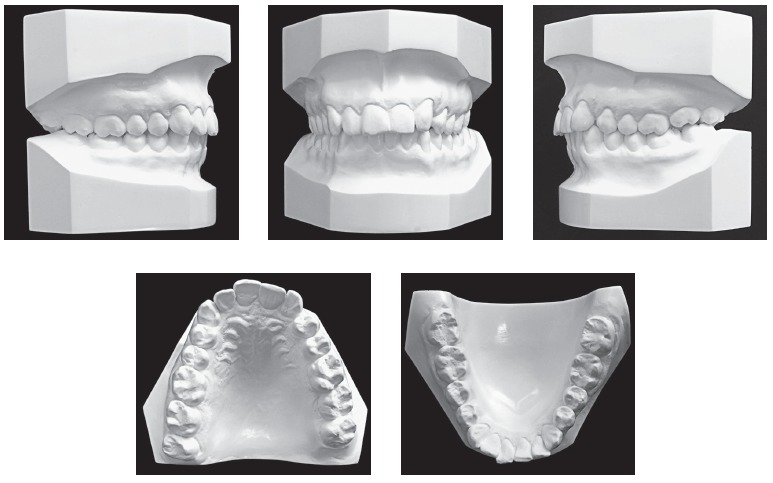
- Initial casts.

As shown in Figure 1, facial analysis revealed a convex profile. The lower third of the face was balanced and associated with a concave profile (UL - S-Line = -2 mm, LL - S-Line = -2 mm), there was passive lip seal, normal nasolabial angle and acute mentolabial angle. Smile analysis revealed maxillary midline deviation to the left, with distinct inclination of the occlusal plane in the anterior region. Dental analysis (Figs 1, 2) revealed Angle Class II, Division 1 malocclusion, mild curve of Spee, 3-mm overbite and 3.5-mm overjet. There was tooth-bone discrepancy of -1.5 mm in the maxilla and -3 mm in the mandible, in addition to absence of tooth #22.

**Figure 3:**
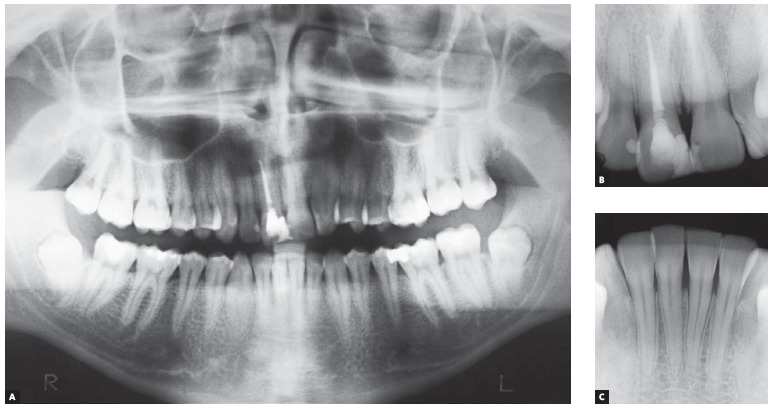
- Initial panoramic (A) and periapical radiographs of maxillary (B) and mandibular (C) incisors.

Panoramic radiograph (Fig 3) revealed that all permanent teeth were present, except for tooth #22. Maxillary third molars were unerupted, whereas mandibular third molars were proclined and impacted at the mandibular ramus. A more thorough analysis performed by means of anterior periapical radiograph evinced mild shortening of tooth #11 root which had been endodontically treated. All remaining incisors had normal root and bone trabeculae contours.

**Figure 4:**
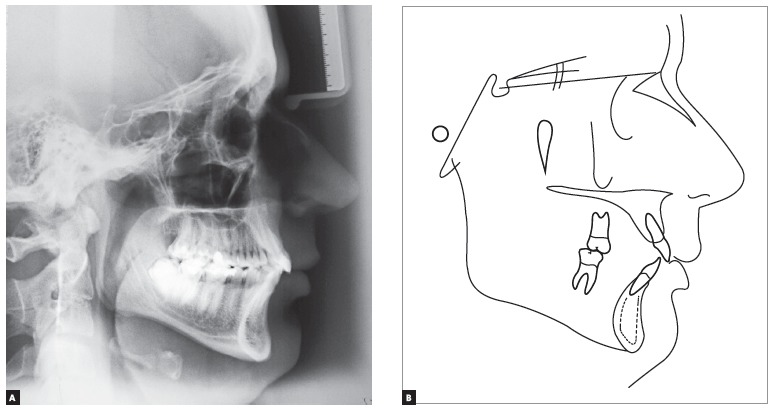
- Initial lateral cephalogram (A) and cephalometric tracing (B).

Cephalometric examination (Fig 4 and Table 1) revealed a Class II skeletal pattern (ANB = 5° and Wits = 3 mm), slight protrusion of the maxilla in relation to the base of the skull (SNA = 84°) and retrusion of the mandible (SNB = 79°) in relation to the maxilla. Vertical pattern analysis revealed decreased angular measurements: SN-GoGn = 27°, FMA = 18° and Y-axis = 56.5°. The patient also presented with discreet labial proclination and protrusion of maxillary incisors (1.NA = 24° and 1-NA = 5 mm) associated with significant labial proclination and protrusion of mandibular incisors (IMPA = 107°, 1.NB = 34°, 1-NB = 7.5 mm and 1-APo = 3.5 mm).

**Table 1:** - Initial (A) and final (B) cephalometric values.

	**Measurements**		**Normal**	**A**	**B**	**Dif. A/B**
Skeletal pattern	SNA	(Steiner)	82°	84°	84°	0
	SNB	(Steiner)	80°	79°	79°	0
	ANB	(Steiner)	2°	5°	5°	0
	Wits	(Jacobson)	F 0 ± 2 mm M 1 ± 2 mm	3 mm	3 mm	0
	Angle of convexity	(Downs)	0°	7.5°	7.5°	0
	Y-axis	(Downs)	59°	56.5°	56.5°	0
	Facial angle	(Downs)	87°	91°	90°	1
	SN-GoGn	(Steiner)	32°	27°	27°	0
	FMA	(Tweed)	25°	18°	18°	0
Dental pattern	IMPA	(Tweed)	90°	107°	97°	10
	1.NA (degrees)	(Steiner)	22°	24°	21°	3
	1-NA (mm)	(Steiner)	4 mm	5 mm	2 mm	3
	1.NB (degrees)	(Steiner)	25°	34°	24°	10
	1-NB (mm)	(Steiner)	4 mm	7.5 mm	5.5 mm	2
	- Interincisal angle	(Downs)	130°	117.5°	130°	12.5
	1-APo	(Ricketts)	1 mm	3.5 mm	1.5 mm	2
Profile	Upper lip - S-line	(Steiner)	0 mm	-2 mm	-1 mm	1
	Lower lip - S-line	(Steiner)	0 mm	-2 mm	-2 mm	0

## TREATMENT PLAN

The main treatment objectives were: correction of sagittal relationship between the maxilla and the mandible; correction of midline deviation, so as to cause maxillary and mandibular midlines to coincide with the facial midline; correction of overbite and leveling of the occlusal plane in the anterior region, so as to create the ideal conditions for esthetic rehabilitation of anterior teeth. Thus, treatment planning included extraction of tooth #14, with distalization of anterior teeth on the right side. The final goal was to achieve Class II molar relationship on both sides, and Class I canine relationship on the right side. On the left side, the first premolar would replace the canine, so as to occlude with teeth #33 and #34. Treatment planning also included full fixed appliance on both arches, with standard Edgewise 0.022 x 0.028-in slots, orthodontic bands on maxillary first molars and mandibular first and second molars, and bonding to the remaining teeth. In the maxilla, enameloplasty and 1-mm interproximal reduction were planned for tooth #23, so as to adjust its shape and achieve lateral guidance. The procedures were followed by extraction of tooth #14. In the mandible, 1.5-mm interproximal reduction was planned for all anterior teeth, so as to aid crowding correction and prevent potential proclination of teeth during alignment and leveling.

Subsequently, space closure of the extraction site would be performed by means of sliding mechanics, associated with correction of the lower curve of Spee achieved with rectangular archwire with reversed curve and torque control in the anterior region. During space closure, a cantilever would be installed on the right side of the maxilla for intrusion of anterior teeth and leveling of the occlusal plane. The moment resulting from the use of a cantilever on tooth #16 would aid anchorage control. Intermaxillary elastics would be used for anchorage control. Correction of midline deviation was also planned.

## TREATMENT PROGRESS

**Figure 5:**
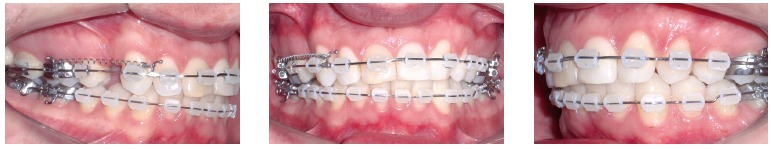
- Intraoral photographs showing sliding mechanics used for space closure after extraction of tooth #14.

Once the full fixed appliance was placed and interproximal reduction was carried out on both arches, alignment and leveling of maxillary and mandibular teeth were performed with superelastic nickel-titanium 0.012-in, 0.014-in and 0.016-in wires followed by stainless steel 0.016-in, 0.018-in and 0.020-in wires. At this phase, a stainless steel 0.019 x 0.026-in archwire was installed in the maxilla with a hook placed distally to tooth #12, with a superelastic nickel-titanium closed loop from tooth #12 to #16, under 300 g of force, for space closure by sliding mechanics (Fig 5). As for the mandible, a stainless steel 0.019 x 0.025-in archwire was manufactured with reverse curve of Spee and buccal root torque to control incisors proclination in the anterior region.

**Figure 6:**
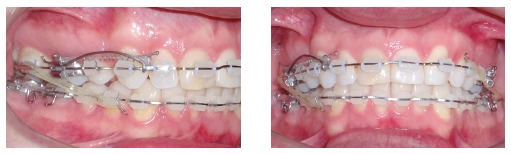
- Intraoral photographs after space closure with a cantilever and intermaxillary elastics concurrently used on the right side.

As planned, during space closure after extraction of tooth #14, a cantilever was placed on the right side of the maxilla for intrusion of anterior teeth and leveling of the occlusal plane. At space closure onset, intermaxillary Class III elastics were used to enhance molar intercuspation on both sides and control buccal proclination of mandibular incisors. By the end of the space closure phase, Class III elastics remained in use on the right side, whereas Class II elastics were the choice for the left side. This was done so in order to enhance intercuspation of the premolar replacing the canine on the left side and to cause maxillary and mandibular midlines to coincide (Fig 6).

Once the aforementioned procedures had been carried out, stainless steel 0.019 x 0.025-in rectangular coordinate archwires were installed under ideal torque. Treatment finishing was performed with 0.018-in archwires associated with single bends, whenever necessary, and intermaxillary elastics 1/8-in in diameter for intercuspation.

Once the active phase of treatment was concluded, the full fixed appliance was removed and a fixed retainer was bonded to mandibular teeth, from canine to canine. A wraparound removable retainer was used in the maxilla. The patient was referred for esthetic treatment, so as to have esthetic rehabilitation of dark teeth, also in need of reshaping, carried out with dental composite.

## RESULTS

**Figure 7:**
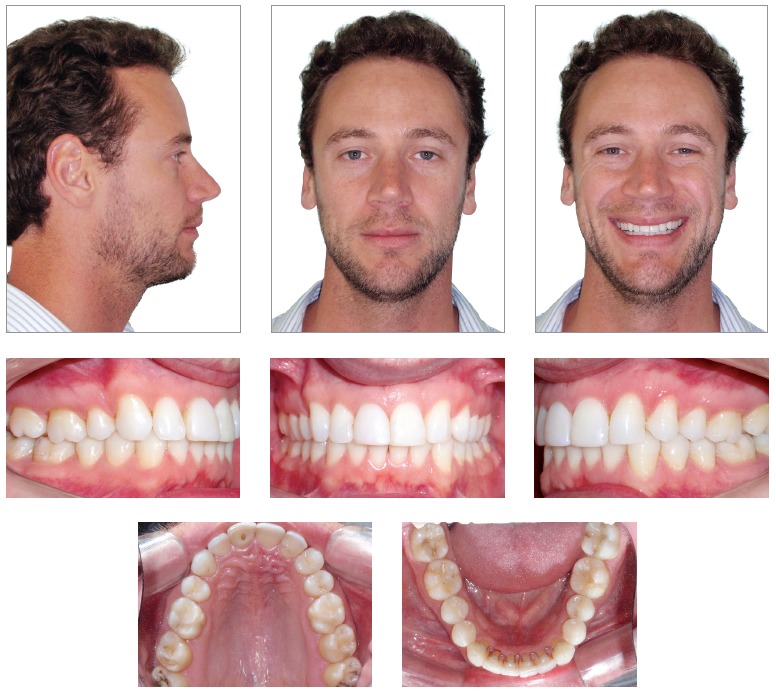
- Final facial and intraoral photographs.

**Figure 8:**
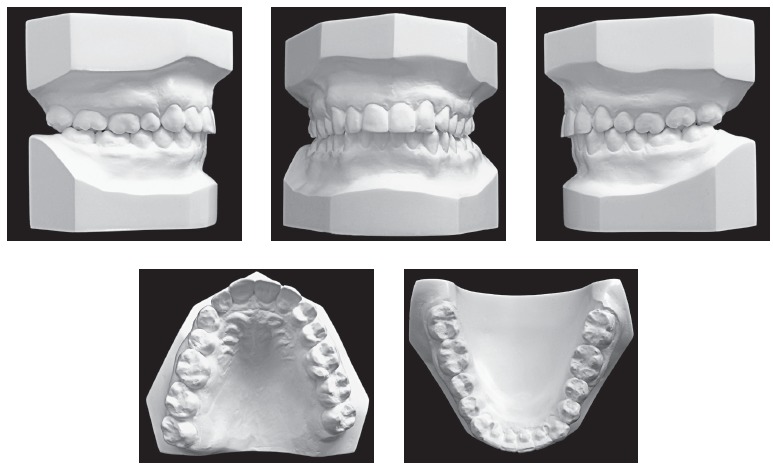
- Final casts.

**Figure 9:**
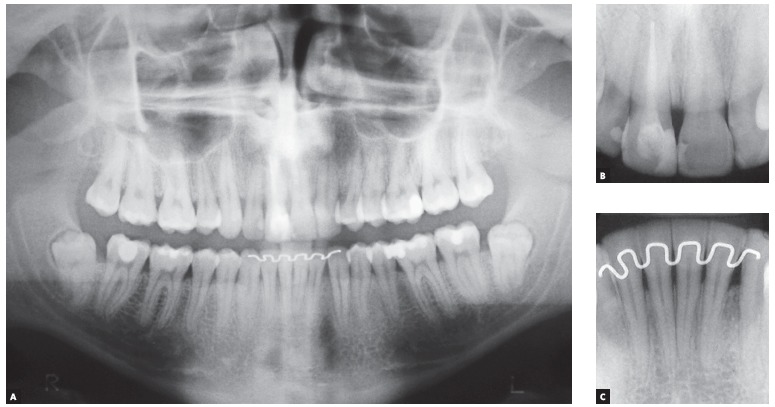
- Final panoramic (A) and periapical radiographs of maxillary (B) and mandibular (C) incisors.

**Figure 10:**
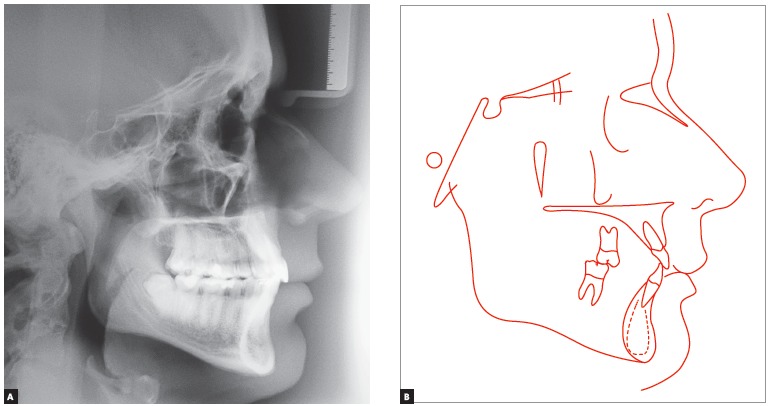
- Final lateral cephalogram (A) and cephalometric tracing (B).

As revealed by final examinations (Figs 7 to 10), patient's facial profile remained without significant alterations after treatment completion. Upper and lower lips remained practically unchanged, with only a minor alteration in the upper lip (UL - S-line went from -2 mm to -1 mm). Improvements in the mentolabial angle were also noted, with preservation of passive lip seal. Smile analysis revealed significant changes in smile harmony as a result of correction of occlusal plane inclination.

Class II molar relationship was satisfactorily achieved on both sides, in addition to Class I canine relationship on the right side and adequate intercuspation on the left side, with maxillary first premolar occluding at canine position. Additionally, the canine replacing the lateral incisor was reshaped and rendered favorably esthetic. Adequate overjet and overbite were also achieved, with satisfactory relationship between the maxilla and the mandible in both vertical and horizontal directions. In terms of function, balanced occlusion was achieved during protrusion and lateral guidance movements on both right and left sides.

Panoramic and periapical radiograph analyses revealed apical roots of maxillary incisors slightly round-shaped, which is consistent with the movements performed (Fig 9). Cephalometric examination (Fig 10 and Table 1) revealed that the sagittal relationship remained stable, with SNA, SNB and ANB angles and Wits value unchanged. Facial height also remained stable, with SN-GoGn, FMA and Y-axis angles unchanged.

**Figure 11:**
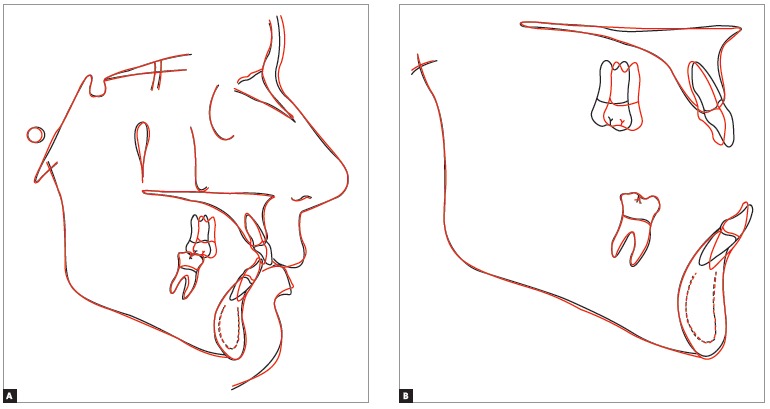
- Total (A) and partial (B) cephalometric superimpositions of initial (black) and final (red) tracings.

Cephalometric superimpositions of initial and final tracings (Fig 11) revealed unchanged vertical dimensions as well as unchanged maxilla and mandible. Facial profile underwent minimal changes, with minor opening of the mentolabial angle. Maxillary partial superimposition revealed that incisors underwent mild retraction and lingual tipping, whereas the right molar was significantly mesially tipped without extrusion. Mandibular superimposition revealed that incisors underwent mild retraction and lingual tipping, whereas molars remained unchanged.

## FINAL CONSIDERATIONS

Epidemiological studies^1^^-^ 6 found a prevalence of agenesis of maxillary lateral incisors varying from 1% to 3%, with genetics most likely representing the major etiological factor.^7 ^In Caucasians, maxillary lateral incisors represent approximately 20% of missing teeth.^8 ^Such an alteration is viewed as an extremely complex issue to be addressed by Orthodontics. There is an increasing need for therapy capable of solving this problem, since this condition has a highly negative impact on facial aesthetics due to lack of continuity resulting from absence of lateral incisors in the maxilla.^9^^-^ 11

As for the case presented herein, it is worth noting the importance of thorough reshaping of the left maxillary canine performed at treatment finishing. The procedure was carried out with a view to rendering this tooth similar to the lateral incisor and achieving group disocclusion on the same side, since the first premolar replaced the canine, both in position and function.^12 ^In the past, some authors^13 ^used to consider canine Class I relationship as key to periodontal health and satisfactory occlusion. However, from the 1950s on, the procedures of canine lateral guidance and mesialization of the first premolar became rather popular, with some studies yielding great results, also in the long term.^8^^,^ 14 ^,^ 15 ^,^ 16

Thus, replacing the lateral incisor by a canine instead of opening space for prosthetic rehabilitation proves to be advantageous, since it yields satisfactory esthetic outcomes, does not induce functional problems to arise at the temporomandibular joint and allows periodontal health conditions to be better maintained, when compared to implant-prosthetic rehabiliation cases.^8^^,^ 14 ^,^ 15 ^,^ 16 Furthermore, although canines might not end up with satisfactory color and shape in some cases, recontouring procedures associated with bleaching and composite resin restoration provides great esthetic results.^10^^,^ 11 ^,^ 12 ^,^ 17

In the present case, only one maxillary right premolar was extracted with a view to correcting maxillary midline deviation and counterbalancing Class II molar relationship with the least esthetic damage possible, while causing minimal changes to patient's lower face. Extracting three premolars (teeth #14, 34 and 44) with a view to counterbalancing agenesis at the upper left hemiarch would invariably lead to retraction of mandibular incisors with potential deterioration of patient's facial profile. Another possibility would be the distalization of posterior teeth on the right side, which would aim at achieving Class I molar relationship on the same side. Nevertheless, this alternative would render the use of Class III elastics to control the position of mandibular incisors impossible, in addition to extending treatment time and requiring extraction of tooth #18 with a view to opening space in the tuberosity for molar distalization on the same side.

Therefore, examinations obtained at treatment completion reveal that all main treatment objectives set at treatment onset were achieved within a total treatment time of 24 months. The occlusal plane was properly leveled in the anterosuperior region, thereby providing the ideal conditions for adequate esthetic rehabilitation in this region.
